# Supplementary material for: Extracellular phase separation mediates storage and release of thyroglobulin in the thyroid follicular lumen
Source: Commun Biol. 2025 Mar 21;8:466. doi: 10.1038/s42003-025-07909-z (PMC11928559; doi:10.1038/s42003-025-07909-z)
Supplement: Supplementary file 1 — Supplementary Information [file 42003_2025_7909_MOESM1_ESM.pdf]

Supplementary Information: Extracellular phase separation mediates storage and release of thyroglobulin in the thyroid follicular lumen

Yihan Yao[1], Nadia Erkamp[1], Tomas Sneideris[1], Xiqiao Yang[1], Rob Scrutton[1], Matthias M. Schneider[1], Charlotte M. Fischer[1], Erik Schoenmakers[2], Nadia Schoenmakers[2], Tuomas P.J. Knowles[1*]

[1]Yusuf Hamied Department of Chemistry, University of Cambridge, Lensfield Road, Cambridge CB2 1EW, United Kingdom

[2] University of Cambridge Metabolic Research Laboratories, Wellcome Trust-MRC Institute of Metabolic Science, University of Cambridge, Cambridge CB2 0QQ, United Kingdom

[*] Correspondence should be addressed to tpjk2@cam.ac.uk

Supplementary Figures

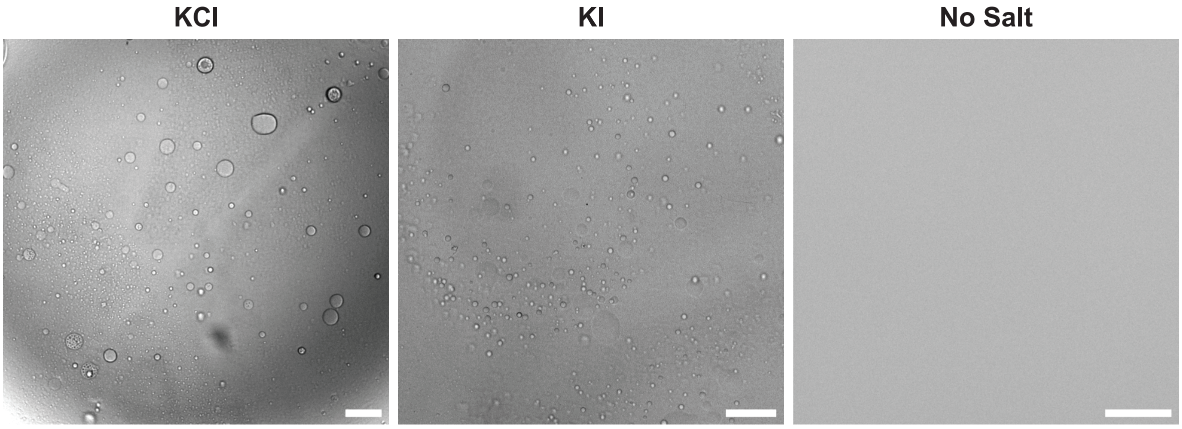

Supplementary Figure 1. Unlabelled Tg forms condensates *in vitro*. Left, unlabelled Tg condensates formed under 250 mM KCl. Middle, unlabelled Tg condensates formed under 250 mM KI. Right, the system remains mixed without adding salt. No Tg condensate formed without salt. All images acquired under bright field. Scale bars, 50 µm. All samples contained 10 µM unlabelled Tg, 4 w/v% PEG 20k in 50 mM HEPES buffer pH=7.5.

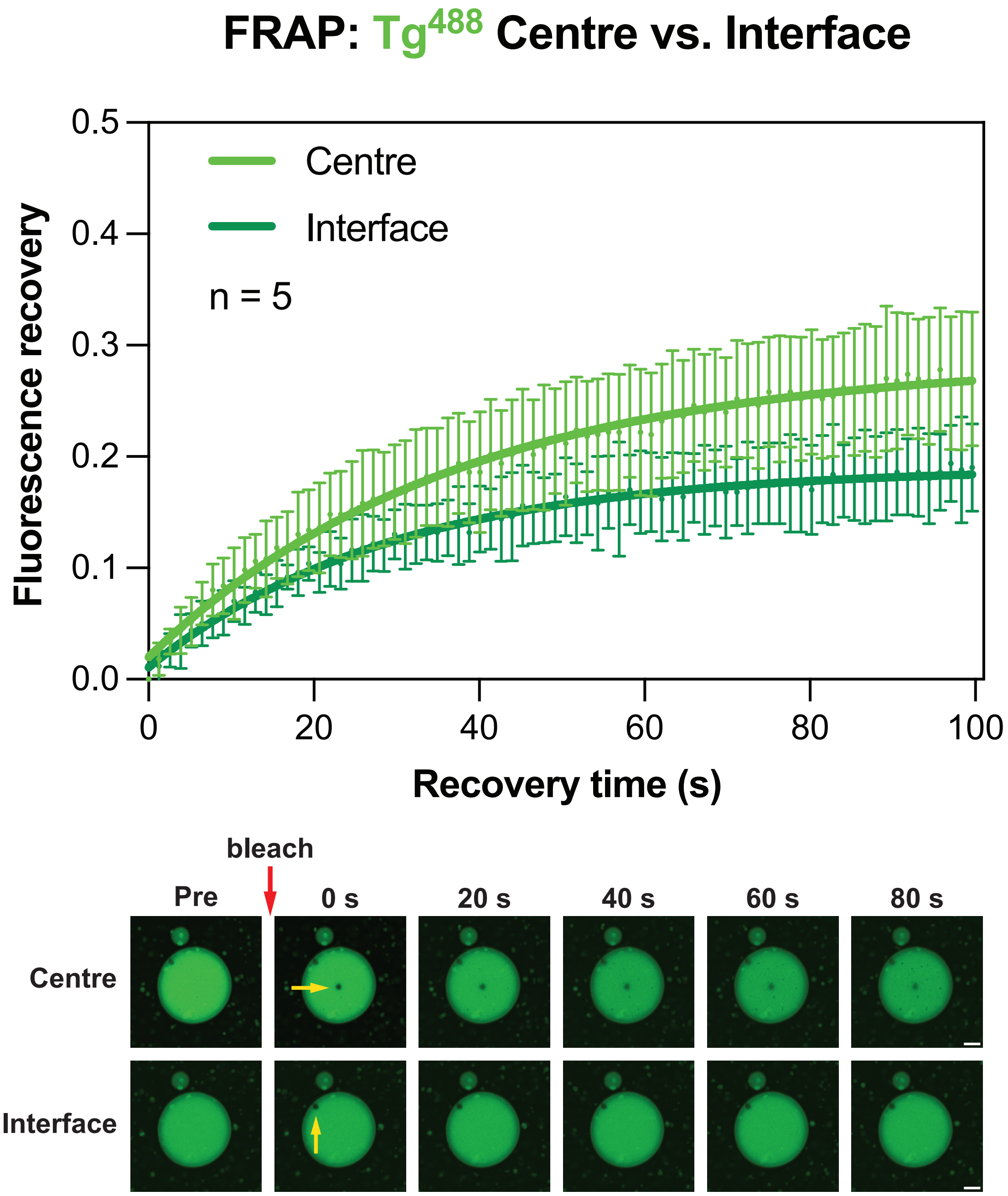

Supplementary Figure 2. The interface and centre of partially aged Tg condensates exhibit distinct dynamics in FRAP analysis. Top: FRAP analysis of areas near the interface and centre of Tg condensates aged at room temperature for approximately 20 minutes. Across all recorded time points, fluorescence recovery was consistently higher at the centre compared to the interface. The curve fitting represents the averaged FRAP data collected from five aged Tg condensates, each bleached at both the interface and centre. In all cases, fluorescence recovery at the centre exceeded that at the interface across all recorded time points. Centre: plateau = 0.29, R2 = 0.72, t1/2 = 25.67 s; interface: plateau = 0.19, R2 = 0.72, t1/2 = 20.42 s. Error bars represent standard deviation. **Bottom:** Confocal images of a representative condensate during FRAP. Scale bars, 10 µm. Condensates were formed at 12.5 µM Tg, 300 mM KCl and 4 w/v% PEG 20k in 50mM, pH=7.5 HEPES buffer.

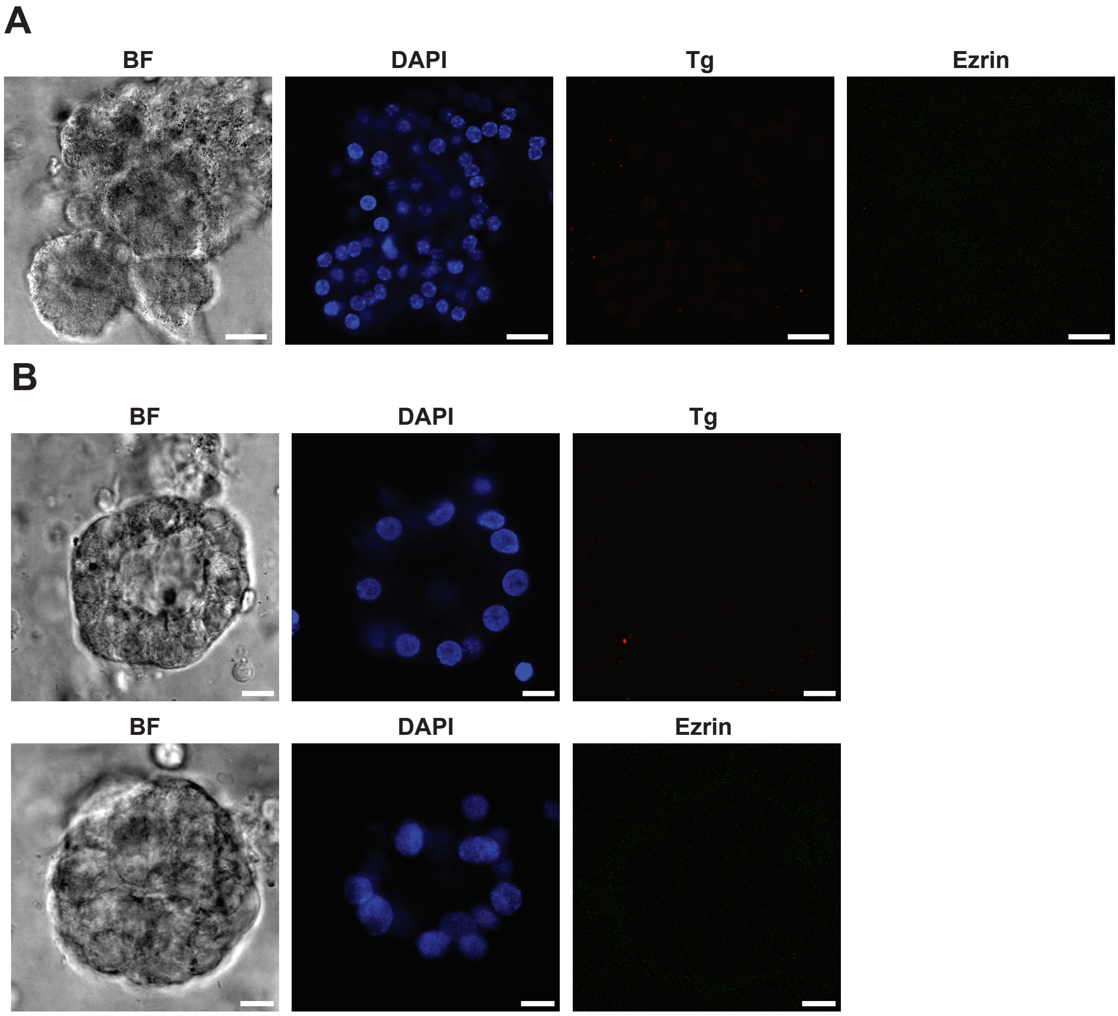

Supplementary Figure 3. Isotype control images of Tg and ezrin in cultured mouse and human thyroid follicles. Confocal images of Tg immunofluorescence isotype control experiment on **(A)** mouse follicles and **(B)** human follicles, imaged under bright field (BF), DAPI (thyrocyte nuclei), AF594 (Tg) and AF488 (ezrin) fluorescence channels. Follicles were observed under bright field; thyrocyte nuclei staining was also revealed under DAPI channel. No Tg or ezrin staining was observed, indicating high specificity of Tg and ezrin primary antibody binding. Scale bars, (A) 20 µm; (B) 10 µm.

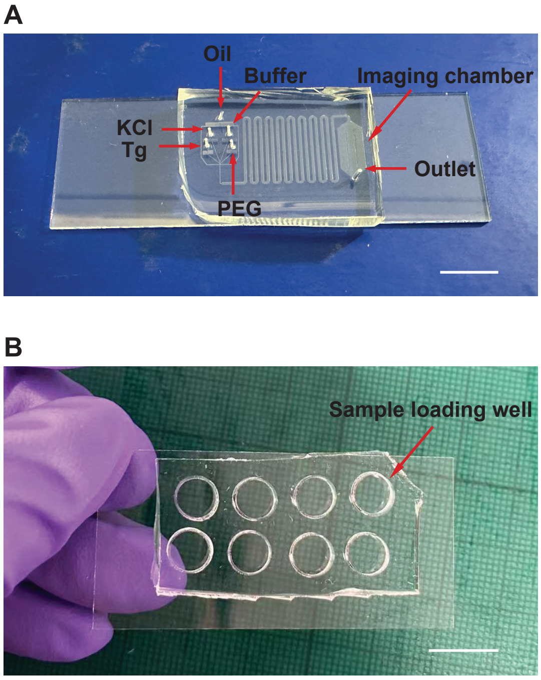

Supplementary Figure 4. PDMS devices for PhaseScan experiments and imaging Tg condensates *in vitro*. **(A)** The PDMS-based microfluidic device used for Tg PhaseScan experiment. This device has a droplet-making part containing 4 inlets for aqueous solutions and 1 inlet for oil; serpentine channels to stabilise oil flow rate and incubate droplets to a thermodynamically stable state before imaging, and an imaging chamber. **(B)** Sample loading well made of PDMS for confocal imaging. The chamber is made by PDMS covalently attached on glass coverslip. Scale bars, 1 cm.
