# Supplementary material for: Extracellular phase separation mediates storage and release of thyroglobulin in the thyroid follicular lumen
Source: Commun Biol. 2025 Mar 21;8:466. doi: 10.1038/s42003-025-07909-z (PMC11928559; doi:10.1038/s42003-025-07909-z)
Supplement: Supplementary file 2 — Description of Additional Supplementary Files [file 42003_2025_7909_MOESM2_ESM.pdf]

Description of Additional Supplementary Files

File name: Supplementary Data 1
Description: The source data behind figures in the main text and supplementary information file.

File name: Supplementary movie 1_condensate merging.avi
Description: Movie of Tg condensate merging illustrated in figure 2C.

File name: Supplementary movie 2_fresh dissolution.avi
Description: Movie of dissolution of fresh Tg condensates illustrated in figure 3A.

File name: Supplementary movie 3_aged dissolution.avi
Description: Movie of dissolution of aged Tg condensates illustrated in figure 3B.
