# Supplementary material for: Extracellular phase separation mediates storage and release of thyroglobulin in the thyroid follicular lumen
Source: Commun Biol. 2025 Mar 21;8:466. doi: 10.1038/s42003-025-07909-z (PMC11928559; doi:10.1038/s42003-025-07909-z)
Supplement: Supplementary file 7 — Reporting summary [file 42003_2025_7909_MOESM7_ESM.pdf]

nature portfolio

Corresponding author(s): Tuomas P.J. Knowles

Last updated by author(s): Feb 25, 2025

Reporting Summary

Nature Portfolio wishes to improve the reproducibility of the work that we publish. This form provides structure for consistency and transparency in reporting. For further information on Nature Portfolio policies, see our Editorial Policies and the Editorial Policy Checklist.

Statistics

For all statistical analyses, confirm that the following items are present in the figure legend, table legend, main text, or Methods section.

n/a	Confirmed	
☐	☒	The exact sample size (*n*) for each experimental group/condition, given as a discrete number and unit of measurement
☐	☒	A statement on whether measurements were taken from distinct samples or whether the same sample was measured repeatedly
☒	☐	The statistical test(s) used AND whether they are one- or two-sided<br>*Only common tests should be described solely by name; describe more complex techniques in the Methods section.*
☒	☐	A description of all covariates tested
☒	☐	A description of any assumptions or corrections, such as tests of normality and adjustment for multiple comparisons
☐	☒	A full description of the statistical parameters including central tendency (e.g. means) or other basic estimates (e.g. regression coefficient) AND variation (e.g. standard deviation) or associated estimates of uncertainty (e.g. confidence intervals)
☒	☐	For null hypothesis testing, the test statistic (e.g. *F*, *t*, *r*) with confidence intervals, effect sizes, degrees of freedom and *P* value noted<br>*Give P values as exact values whenever suitable.*
☒	☐	For Bayesian analysis, information on the choice of priors and Markov chain Monte Carlo settings
☒	☐	For hierarchical and complex designs, identification of the appropriate level for tests and full reporting of outcomes
☒	☐	Estimates of effect sizes (e.g. Cohen's *d*, Pearson's *r*), indicating how they were calculated

Our web collection on statistics for biologists contains articles on many of the points above.

Software and code

Policy information about availability of computer code

Data collection	Las X, inbuilt Leica Stellaris 5 software; Micro-Manager 2.0.0
Data analysis	ImageJ2 (version 2.14.0/1.54f); Python 3.9.7; Microsoft Excel 365; GraphPad Prism (Version 10.4.1); Python-based combinatorial microdroplet analysis script (PhaseScan): https://github.com/rqi14/PhaseScan

For manuscripts utilizing custom algorithms or software that are central to the research but not yet described in published literature, software must be made available to editors and reviewers. We strongly encourage code deposition in a community repository (e.g. GitHub). See the Nature Portfolio guidelines for submitting code & software for further information.

Data

Policy information about availability of data

All manuscripts must include a data availability statement. This statement should provide the following information, where applicable:

- Accession codes, unique identifiers, or web links for publicly available datasets
- A description of any restrictions on data availability
- For clinical datasets or third party data, please ensure that the statement adheres to our policy

All data generated or analyzed during this study are included in this article and its supplementary information files. Original images and data will be made available upon request. Source data are provided with this paper.

Research involving human participants, their data, or biological material

Policy information about studies with human participants or human data. See also policy information about sex, gender (identity/presentation), and sexual orientation and race, ethnicity and racism.

Reporting on sex and gender	This study reported analyses from 2 thyroidectomy samples from 2 female patients.
Reporting on race, ethnicity, or other socially relevant groupings	Not available
Population characteristics	Human research participants were individuals undergoing thyroidectomy for routine clinical indications (Graves' disease) who consented to the use of surplus tissue for research purposes.
Recruitment	Participants were recruited from clinics at Addenbrooke's Hospital, Cambridge, United Kingdom.
Ethics oversight	The study was performed using the ethically-approved protocol 21/SC/0158 by South Central Oxford B REC.

Note that full information on the approval of the study protocol must also be provided in the manuscript.

Field-specific reporting

Please select the one below that is the best fit for your research. If you are not sure, read the appropriate sections before making your selection.

☒ Life sciences ☐ Behavioural & social sciences ☐ Ecological, evolutionary & environmental sciences

For a reference copy of the document with all sections, see nature.com/documents/nr-reporting-summary-flat.pdf

Life sciences study design

All studies must disclose on these points even when the disclosure is negative.

Sample size	No sample-size calculation was performed. Three independent sets of cultured primary mouse thyroid follicles and two independent sets of cultured primary human thyroid follicles were used for ex vivo immunofluorescence experiments to show that similar results were observed in biological replicates.
Data exclusions	No data was excluded from the analyses.
Replication	Where possible, experiments were replicated at least three times to ensure reproducibility.
Randomization	Not relevant to this study, no samples/organisms/participants were allocated into experimental groups.
Blinding	Not relevant to this study, no group allocation was performed during data collection and/or analysis.

Reporting for specific materials, systems and methods

We require information from authors about some types of materials, experimental systems and methods used in many studies. Here, indicate whether each material, system or method listed is relevant to your study. If you are not sure if a list item applies to your research, read the appropriate section before selecting a response.

Materials & experimental systems

n/a	Involved in the study
☐	☒ Antibodies
☒	☐ Eukaryotic cell lines
☒	☐ Palaeontology and archaeology
☐	☒ Animals and other organisms
☒	☐ Clinical data
☒	☐ Dual use research of concern
☒	☐ Plants

Methods

n/a	Involved in the study
☒	☐ ChIP-seq
☒	☐ Flow cytometry
☒	☐ MRI-based neuroimaging

Antibodies

Antibodies used	anti-thyroglobulin Abcam Ab156008; anti-Ezrin, Invitrogen clone 3C12; Abcam anti-rabbit IgG 172730; Invitrogen anti-mouse IgG1

Antibodies used	kappa 14-4714-81; Abcam Donkey pAb to Rabbit IgG Fluor 594, Ab150064; Invitrogen donkey anti-mouse Alexa Fluor 488 A21202
Validation	Isotype control experiments shown in Supplementary Figure 3 validate the specificity of primary antibodies used in this study.

Animals and other research organisms

Policy information about studies involving animals; ARRIVE guidelines recommended for reporting animal research, and Sex and Gender in Research

Laboratory animals	The study used pooled samples from C57BL/6 mice, n=10 females, n=1 male, aged 5-47 weeks.
Wild animals	The study did not involve wild animals.
Reporting on sex	Samples were pooled and included predominantly female samples and one male sample as above.
Field-collected samples	The study did not involve samples collected from the field.
Ethics oversight	Animals were housed in individually ventilated cages (IVCs) in an environmentally controlled room, temperature 22°C (+/-2°C) and 55%RH (+/- 10%), with a 12/12-h light/dark cycle, and ad libitum access to food and water. Thyroid tissue was obtained from surplus wild-type mice (C57BL/6) bred under different protocols regulated under the Animals (Scientific Procedures) Act 1986 Amendment Regulations 2012 following ethical review by the University of Cambridge Animal Welfare and Ethical Review Body and shared amongst facility users according to 3Rs. Mice were culled humanely using a rising concentration of CO_2.

Note that full information on the approval of the study protocol must also be provided in the manuscript.

Plants

Seed stocks	*Report on the source of all seed stocks or other plant material used. If applicable, state the seed stock centre and catalogue number. If plant specimens were collected from the field, describe the collection location, date and sampling procedures.*
Novel plant genotypes	*Describe the methods by which all novel plant genotypes were produced. This includes those generated by transgenic approaches, gene editing, chemical/radiation-based mutagenesis and hybridization. For transgenic lines, describe the transformation method, the number of independent lines analyzed and the generation upon which experiments were performed. For gene-edited lines, describe the editor used, the endogenous sequence targeted for editing, the targeting guide RNA sequence (if applicable) and how the editor was applied.*
Authentication	*Describe any authentication procedures for each seed stock used or novel genotype generated. Describe any experiments used to assess the effect of a mutation and, where applicable, how potential secondary effects (e.g. second site T-DNA insertions, mosiacism, off-target gene editing) were examined.*
